# Epidemiologic trends of infants with orofacial clefts in a multiethnic country: a retrospective population-based study

**DOI:** 10.1038/s41598-021-87229-4

**Published:** 2021-04-06

**Authors:** Mimi Yow, Aizhen Jin, George Seow Heong Yeo

**Affiliations:** 1 Department of Orthodontics, National Dental Centre Singapore, Second Hospital Avenue, Singapore, 168938 Singapore; 2 Department of Dental Medicine, Karolinska Institutet, Alfred Nobels Allé 8, Huddinge, Sweden; 3 Centre for Healthy Longevity, Yong Loo Lin School of Medicine, National University of Singapore, 10 Medical Drive, Singapore, 117597 Singapore; 4 Department of Maternal and Fetal Medicine, KK Women’s and Children’s Hospital, 100 Bukit Timah Road, Singapore, 229899 Singapore

**Keywords:** Developmental biology, Diseases, Medical research

## Abstract

Cleft births surveillance is essential in healthcare and prevention planning. Data are needed in precision medicine to target upstream management for at-risk individuals. This study characterizes Singapore’s population-based orofacial cleft topography by ethnicity and gender, and establishes the cleft cohort’s infant mortality rate. Data, in the decade 2003 to 2012, were extracted by the National Birth Defects Registry. Trend testing by linear regression was at p < 0.05 significance level. Prevalence per 10,000 for population-based cleft live births was 16.72 with no significant upward trend (p = 0.317). Prevalence rates were 8.77 in the isolated cleft group, 7.04 in the non-isolated cleft group, and 0.91 in the syndromic cleft group. There was significant upward trend in infants with non-isolated clefts (p = 0.0287). There were no significant upward trends in infants with isolated clefts and syndromic clefts. Prevalence rates were sexually dimorphic and ethnic-specific: male 17.72; female 15.78; Chinese group 17.17; Malay group 16.92; Indian group 10.74; and mixed ethnic origins group 21.73. The overall infant mortality rate (IMR) was 4.8% in the cohort of 608 cleft births, which was more than double the population-based IMR of 2.1% in the same period. Infants with non-isolated and syndromic clefts accounted for 96.6% of the deaths.

## Introduction

Orofacial clefts (OFC) are common birth defects with wide-ranging prevalence in different parts of the world^1^. There is much heterogeneity in OFC birth defects and the aetiologies of the various cleft-types are distinct involving different embryological origins and timing of development^2,3^. Laterality is featured in individuals with OFC and unilateral left-sided findings are common^4^. Identification of the different subphenotypes and cleft laterality is important in understanding the genetics, epigenetics, and environmental factors to identify individuals at risk and triggers^5^.

Comparisons of neonatal mortality rate (NMR) and infant mortality rate (IMR) of infants, with and without associated anomalies, revealed higher mortality rates for the latter compared to the population norm^6^. Long-term survival of individuals with OFC was reduced^7^. They had poorer health and shorter life spans^8^. These indicated the need to address their life course health concerns. The key issues are to identify the patterns attributable to modifiable or non-modifiable factors for future treatment and prevention. The first step to change the corollary is data tracking to understand the at-risk groups susceptible to OFC pregnancies. Well-informed decisions are all-important in health policies to drive prevention programs. In upstream healthcare for prevention, detailed cleft-births surveillance and data of affected individuals, families, ethnic groups, locations and environment are essential.

The objectives of this study were to establish the resident population-based prevalence and trends of live cleft births in Singapore from 2003 to 2012, delineate the ethnic and gender-specific features, and establish the associated congenital malformations and the IMR. The groups studied were:

- 1. Isolated cleft—infants with only orofacial cleft defects and no other malformations^5^.
- 2. Non-isolated cleft—infants with orofacial cleft defects and other congenital malformations with no consistent pattern that could be defined as a sequence or a syndrome^9^.
- 3. Syndromic cleft—infants with orofacial cleft defects with a consistent pattern of co-occurring malformations that did not represent a sequence but were pathogenetically related and defined as a syndrome^10^.

## Results

**Figure 1:**
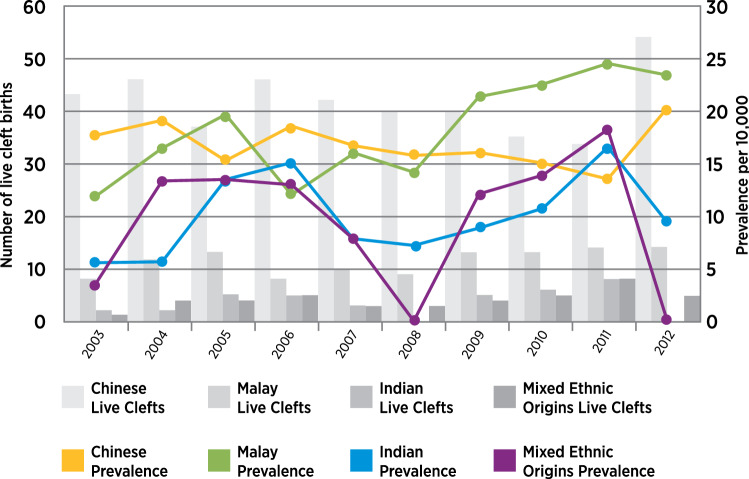
Population-based live cleft births by ethnic groups. Chinese group 17.17 per 10,000. Upward trend is not significant (p = 0.692). Malay group 16.92 per 10,000. Upward trend is significant (p = 0.008). Indian group 10.74 per 10,000. Upward trend is not significant (p = 0.724). Mixed ethnic origins group 21.73 per 10,000. Upward trend is not significant (p = 0.606).

**Figure 2:**
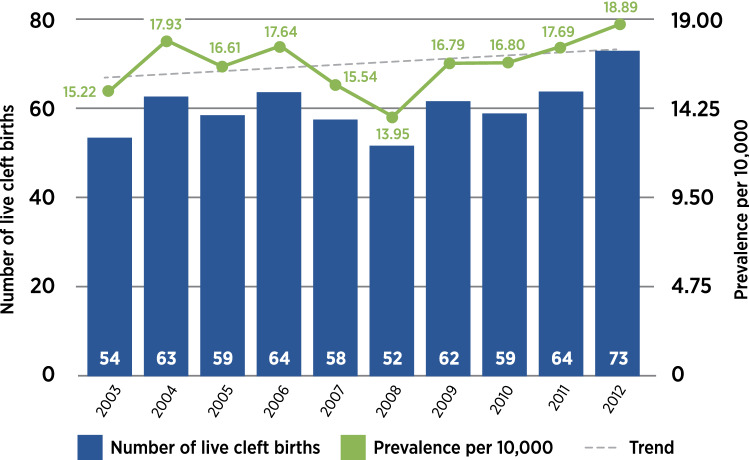
Prevalence of population-based live cleft births by year. Overall prevalence is 16.72 per 10,000. Upward trend is not significant (p = 0.317).

In the period from 2003 to 2012, the population’s multiethnic live births comprised 242,414 (66.7%) Chinese, 67,630 (18.6%) Malay, 33,373 (9.2%) Indian, and 20,216 (5.6%) in the mixed ethnic origins group. The total number of population live births was 363,633, of which 608 were live cleft births comprising 312 males and 296 females. There were 249 infants with cleft palate only (CPO), 115 infants with cleft lip only (CLO), and 244 infants with cleft lip and palate (CLP). The prevalence and trend of live cleft births in the ethnic groups are in Fig. 1. Annualized prevalence and trends are in Fig. 2.

### Cleft-type and prevalence

The distribution of cleft-types is in Table 1. The cleft-types were 41.0% (N = 249) cleft palate only (CPO), 40.1% (N = 244) cleft lip and palate (CLP), and 18.9% (N = 115) cleft lip only (CLO). The prevalence rates per 10,000 for all types of cleft live births were:

**Table 1:** Live births by cleft-type and gender.

Type of cleft		Live births		
		Male	Female	Total
Cleft palate only		97	152	249
Cleft lip only	Unilateral	49	34	83
	Bilateral	9	4	13
	NOS	14	5	19
Cleft lip and palate	Unilateral	86	58	144
	Bilateral	39	26	65
	NOS	18	17	35
Total		312	296	608

*NOS* not otherwise specified (non-classifiable cleft sub-type in ICD 9 & 10).

Isolated cleft—3.00 CPO (N = 109); 1.84 CLO (N = 67); 3.93 CLP (N = 143); 8.77 overall (N = 319).

Non-isolated cleft—3.44 CPO (N = 125); 1.13 CLO (N = 41); 2.48 CLP (N = 90); 7.04 overall (N = 256).

Syndromic cleft—0.41 CPO (N = 15); 0.19 CLO (N = 7); 0.30 CLP (N = 11); 0.91 overall (N = 33).

There was no significant upward trend in infants with isolated clefts (p = 0.0883) and syndromic clefts (p = 0.1908). There was significant upward trend of infants with non-isolated clefts (p = 0.0287). There was no significant overall upward trend in the population-based prevalence of OFC (p = 0.317).

### Cleft-type and gender

Table 1 shows gender distribution for the different cleft-types. There was sexual dimorphism in the overall cleft prevalence per 10,000: 17.72 in males, and 15.78 in females.

The CPO group consisted of 39% (N = 97) males and 61.0% (N = 152) females; the CLP group comprised 58.6% (N = 143) males and 41.4% (N = 101) females; and the CLO group comprised 62.6% (N = 72) males and 37.4% (N = 43) females. The CLP sub-types comprised 59.0% (N = 144) unilateral, 26.6% (N = 65) bilateral, and 14.3% (N = 35) were not otherwise specified (NOS). The CLO sub-types comprised 72.2% (N = 83) unilateral, 11.3% (N = 13) bilateral, and 16.5% (N = 19) NOS.

### Cleft-type and ethnicity

Table 2 shows the ethnic group distribution for the cleft sub-types. The CPO group consisted of 72.3% (N = 180) Chinese, 15.3% (N = 38) Malay, 4.8% (N = 12) Indian, and 7.6% (N = 19) mixed ethnic origins. The CLP group consisted of 65.6% (N = 160) Chinese, 20.9% (N = 51) Malay, 6.6% (N = 16) Indian, and 7.0% (N = 17) mixed ethnic origins. The CLO group consisted of 67.0% (N = 77) Chinese, 20.9% (N = 24) Malay, 7.0% (N = 8) Indian, and 5.2% (N = 6) mixed ethnic origins. The prevalence rates per 10,000 were: Chinese group 17.17, Malay group 16.92, Indian group 10.74, and mixed ethnic origins group 21.73.

**Table 2:** Ethnic groups and cleft-types.

Type of cleft		Infants with isolated clefts				Infants with non-isolated clefts				Infants with syndromic clefts				Total
		C	M	I	O	C	M	I	O	C	M	I	O	
Cleft palate only		73	17	5	14	96	19	6	4	11	2	1	1	249
Cleft lip only	Unilateral	42	5	1	2	14	8	6	3	1	1	0	0	83
	Bilateral	3	3	0	0	3	0	0	0	2	2	0	0	13
	NOS	9	2	0	0	3	3	1	0	0	0	0	1	19
Cleft lip and palate	Unilateral	65	10	5	7	31	12	6	6	1	1	0	0	144
	Bilateral	17	10	2	1	17	9	1	2	5	1	0	0	65
	NOS	21	5	0	0	2	2	2	0	1	1	0	1	35
Total		230	52	13	24	166	53	22	15	21	8	1	3	608

*C* Chinese group, *M* Malay group, *I* Indian group, *O* mixed ethnic origins group, *NOS* not otherwise specified (non-classifiable cleft sub-type in ICD 9 & 10).

### Cleft-associated malformations and syndromes

The distribution of non-isolated and syndromic clefts is in Table 2. Infants with non-isolated clefts comprised 42.1% (N = 256) out of 608 live cleft births. The total number of cleft-associated malformations was 864 and the top ten co-occurring malformations were 31.5% (N = 272) heart anomalies, 24.2% (N = 209) musculoskeletal system anomalies, 9.0% (N = 78) ear, face and neck anomalies (EFN), 7.2% (N = 62) central nervous system anomalies, 5.7% (N = 49) lung anomalies, 4.5% (N = 39) gut anomalies, 4.4% (N = 38) integument anomalies, 4.2% (N = 36) eye anomalies, 2.9% (N = 25) genital anomalies, and 2.6% (N = 22) urinary system anomalies. Infants with syndromic clefts comprised 5.4% (N = 33) of cleft live births. The syndromes were Patau syndrome (N = 9), Edward syndrome (N = 5), and Down syndrome (N = 1).

### Infant mortality

The mortality rate was the number of deaths within the cleft cohort of 608 live cleft-births. The neonatal mortality rate (NMR) was death reported before 28 days and infant mortality rate (IMR) was death reported before 365 days. The overall NMR was 23 per 1000 (2.3%) (N = 14). The overall IMR of isolated, non-isolated, and syndromic clefts was 48 per 1000 (4.8%) (N = 29). The IMR in the isolated cleft group was 1.6 per 1000 (0.16%) (N = 1), the non-isolated cleft group was 25 per 1000 (2.5%) (N = 15), and the combined isolated and non-isolated cleft group was 26 per 1000 (2.6%) (N = 16). The IMR of the syndromic cleft group was 21 per 1,000 (2.1%) (N = 13). The NMR and IMR of the general population were 1 per 1000 (0.1%) and 21 per 1,000 (2.1%), respectively.

## Discussion

### Population prevalence

The population-based prevalence for live cleft births of the resident population, without stillbirths and abortuses, was 16.7 per 10,000 and no significant upward trend (p = 0.317). The population-based prevalence in the previous decade, from 1993 to 2002, was 18.7 per 10 000 with significant upward trend that included stillbirths and abortuses of residents, non-residents, and foreigners in Singapore^11^. The prevalence rates in both decades were above the global average of 9.92 per 10,000 (range 2.89 to 23.85)^1^.

### Ethnic-specific prevalence

Singapore has a multiethnic population constituted by immigrants from the neighbouring countries of China, India and Malaysia. Among the four groups of different ancestral origins in Singapore, the mixed ethnic origins group had the highest prevalence of 21.73 per 10,000 in live cleft births. There were also prevalence differences in the groups with Chinese, Malay and Indian ancestries: 17.17, 16.92, and 10.74 per 10,000, respectively. A similar pattern of descending rates of prevalence in the ethnic groups was present in the previous decade from 1993 to 2002^11^.

The present study found the prevalence of Chinese Singaporeans to be high, 17.17 per 10,000, which was close to the reported prevalence for the ethnic Chinese in China of 16.63 per 10,000^12^. From cross-sectional studies of Indians across India, the pooled prevalence was 13.0 per 10,000^13^. In comparison, the prevalence of Indian Singaporeans was lower, 10.74 per 10,000. Population cohorts of consecutive cleft live births for Indian Singaporeans (N = 36) as well as the group of mixed ethnic origins (N = 42) in this study were small and not representative of these two groups from South Asia and Southeast Asia. The Malay Singaporeans’ prevalence of 16.92 per 10,000 in this study approximated the prevalence of 18.5 per 10,000 of the Malay group reported in northwest Malaysia in a hospital-based study^14^, which would explain the latter’s higher rate than a population-based prevalence.

Variations in ethnic group prevalence could be due to different reasons as population demographics could have changed due to migratory patterns in the past decade. Differences in study designs and sampling i.e., population-based, hospital-based or treatment centre samplings, inclusion and exclusion criteria, different nominators and denominators in determining prevalence, early or late registration of subjects with OFC, were among many reasons that could change prevalence values. The prevalence of the groups with OFC of different ancestries in this study were almost similar to their forebears in the old countries. This corroborated with findings linking ethnic-specific prevalence of OFC to ancestral genes^15^.

### Gender

The male and female live cleft births were 17.72 and 15.78 per 10,000, respectively, and upward trends were not significant for males (p = 0.650) and females (p = 0.294). Distribution by gender was 1.1:1.0 male to female ratio. Gender-specific cleft-types of CLO and CLP were 1.7:1.0 and 1.4:1.0, respectively, with a predisposition for males. This was reversed in CPO with a 1.6:1.0 female to male ratio. Male infants tended to present with CLO and CLP phenotypes and females with the CPO phenotype. The role of gender in different cleft phenotypes was postulated to be due to timing differences in embryological development. Palatal development was slower in females than males, which increased the risk of secondary palate maldevelopment or CPO in females^16^. Gender represented a substantial estimated population attributable fraction in the non-modifiable factors of risk of occurrence for OFC^17^.

### Cleft-types

Cleft palate defects were the most common cleft-type among live cleft births in this study. Distribution by cleft-type was 2.2:2.1:1.0 for CPO, CLP and CLO, respectively. The Global Registry and Database on Craniofacial Anomalies reported findings of Asian OFC registries with low CPO prevalence and high CLP rates; CLP to CPO were 4 to 6 times higher in Asia^18^. In this study, The prevalence for CPO and CLP in our country was almost similar, 6.86 and 6.71 per 10,000, respectively. This could be a reflection of the pooling effect of the multiethnic variations in cleft-types. The multifactorial threshold (MFT) model predicted greater genetic liability within a population with higher overall cleft prevalence that predisposed to greater occurrence of severe cleft types^19^. There were suggestions of CLO and CLP as defects of different severities in the same aetiological spectrum but the evidence was not conclusive. The two entities of CPO and CLP were suggested to be of different aetiological origins and classified separately^20^. In this population with high overall cleft prevalence, there should be a greater number of cases with increased cleft defect severity. However, unilateral CLP occurred four times more often than bilateral CLP. Even in countries with low overall prevalence of less than 10 per 10,000, subjects with CLP were more common than those with CLO. Infants with syndromic clefts in this study were mostly associated with the more severe phenotype of bilateral CLP and CPO.

### Cleft-associated malformations

The number of non-isolated clefts in this study was high and constituted 42.1% of live cleft births. They accounted for almost half (47.5%) of live cleft births when grouped with syndromic clefts (5.4%). Prevalence of non-isolated clefts with associated malformations was 7.04 per 10,000 with a rising trend (p = 0.0287). This was probably due to increased detection and better reporting. Syndromic clefts were also commonly associated with CPO defects (55.6%) and half of infants with CPO (50.2%) were associated with other malformations. The findings concurred with other reports on the high frequency of associated anomalies with CPO cases that should be routinely examined for additional malformations^21–24^. The associated anomalies of infants with CPO in this study involved mostly musculoskeletal system and heart defects. In infants with non-isolated CLO and CLP, associated anomalies were twice more frequent in the latter and involved heart anomalies. The IPDTOC Working Group^1^ reported 15.9% and 7.3% of individuals with clefts had other malformations and syndromes, respectively.

### Neonatal and infant mortality rates

Neonatal and infant death registrations by the Singapore Registry of Births and Deaths were of infants who lived less than 28 days and 365 days, respectively. The neonatal mortality rate (NMR) and IMR of the general population were 1 per 1000 (0.1%) and 21 per 1000 (2.1%)^25^, respectively. The NMR of infants with clefts was 23 per 1000 (2.3%) and the IMR of infants with all cleft-types was 48 per 1000 (4.8%), which more than doubled the population’s NMR and IMR. Infants with clefts that were associated with malformations and syndromic clefts formed 96.6% of infant deaths. There was one death involving an infant with isolated CPO, which could have been a missed diagnosis of a cleft birth with an associated anomaly or an infant with Pierre Robin Sequence. Infants with clefts who perished comprised 49.4% with CLP, 37.3% with CLO, and 13.3% with CPO.

In other developed countries, infants with clefts were also reported as having higher IMR than the general population. In a Danish population study of 678 consecutive live births of infants with OFC from 1977 to 1981, the mortality rate was 35 per 1000 (3.5%) (N = 24) before the age of 22 months^4^. The mortality rate of cleft infants in Denmark was higher compared to the general population’s post-neonatal mortality rate of 30 to 32 per 1000 (3.0 to 3.2%) reported in the same period^26^. A study of Dutch infants with isolated and non-isolated orofacial clefts reported the IMR to be elevated at 21 per 1000 (2.1%), which was significantly higher than the Dutch population IMR of 4.5 per 1000 (0.45%)^27^. In another report by the East of England Cleft Network of 638 children with clefts, the causes of deaths were reported to be from associated anomalies (61%) and infections (17%), with an exceptionally high mortality rate for CPO at 68.1 per 1000 (6.8%). The East of England Cleft Network reported an overall IMR of 36 per 1000 (3.6%) for infants with isolated clefts compared to the IMR in the general population of 4.1 per 1000 (0.4%)^28^.

The reported IMR for all clefts was consistently higher than that of the general population in most reports. The higher infant mortality rates indicated the need for thorough medical examination for associated anomalies and syndromes that could have fatal consequences of infants with OFC.

### Study limitations

The main limitations in this study were the coding systems in force, the ICD-9 and ICD-10, the early registrations that were done at or soon after birth, and no histories of family members with OFC. The registry lacked registration data in maternal, paternal and family histories of OFC to link heredity to affected pregnancy outcomes as cleft births had strong recurrence in first-degree relatives^29^. The ICD-9 and ICD-10 coding systems in force were not structured to record heterogeneity of OFC subphenotypes. This limitation was faced by other researchers as well and there was a call for revision of the ICD coding system to include classification of different OFC sub-types and possible aetiologies^30^. The call was heeded in the newly launched ICD-11^31^ with reorganized classifications for registration of heterogeneous OFC conditions in the new ICD-11 chapter on Developmental Anomalies. In future, isolated structural developmental anomalies, multiple developmental anomalies and syndromes classifications could be augmented by inclusions for molecular genetic or cytogenetic aetiologies with added flexibility for reporting granular details in complex OFC subphenotypes.

The findings in this study are based on diagnostic data soon after birth. Under-reporting of live cleft births and associated malformations is likely and due to early registrations. Cleft uvula and submucosal clefts of the primary and secondary palates are challenging to detect by visual inspection and easily missed in small neonates. Cleft-associated developmental malformations may take time to manifest and often missed in infants with OFC under one year of age. Missed diagnoses with under-detection of associated anomalies may be the causes of infant mortality or lifelong chronic health problems that increased the burden of care and reduced quality of life.

Our team proposes a lifelong open-date registration of individuals with OFC. Continual data updates to the registry will minimise missed diagnoses and improve surveillance accuracy.

## Conclusions

Population-based live cleft birth prevalence was 16.72 per 10,000 with sexual dimorphism, 17.72 and 15.78 per 10,000 in males and females, respectively. There was no significant upward trend of population-based live cleft births. Prevalence per 10,000 varied in the different cleft-types: 8.77 in the isolated cleft group, 7.04 in the non-isolated cleft, and 0.91 in the syndromic cleft group. The prevalence of OFC live cleft births was ethnic-specific, which were 17.17, 16.92, 10.74, and 21.73 per 10,000 in the Chinese, Malay, Indian, and mixed ethnic origins groups, respectively. There was significant upward trend in live cleft births with associated malformations that involved almost half of all live cleft births. The IMR of infants with clefts was 48 per 1000 (4.8%) for all cleft-types, which was more than double the IMR of the population in the same period. Early mortality occurred in non-isolated infants with associated malformations and syndromes, the NMR was 23 per 1000 (2.3%). This underscored the importance of advocating cleft-associated pregnancies for early detection of associated anomalies and hospital births with high-risk infant care facilities.

More importantly, the findings should raise the awareness of and the approach to different ethnic groups with pregnancy outcomes associated with orofacial clefts. As orofacial malformations occur in early pregnancy, surveillance data are important in decisions that target upstream management for prevention of affected births in individuals of at-risk groups.

## Materials and method

This study was approved by the Singapore National Birth Defects Registry Office (Reference: Y15-007) and the SingHealth Centralized Institutional Review Board D (CIRB D Reference: 2014/2199).

### Study population

The reporting of births and deaths in the country are mandated by the Singapore Statutes. The population data sources comprised government organizations, public and private healthcare institutions. The method of data collection by the Singapore National Birth Defects Registry (NBDR) was previously described^32^. The NBDR data from 2003 to 2012 was established from multiple sources: cytogenetics and pathology laboratories, neonatal wards and maternity hospitals, Medi-claims, birth defects, death certificates with reported congenital anomalies, stillbirths and abortuses (spontaneous and elective). The population-based data were extracted and anonymized by the NBDR for this study.

The NBDR system was purpose-built for electronic data capture with case-entry matching against existing records in the system. The merging functions and contradiction modules checked, verified, and handled inconsistencies to resolve discrepancies and duplication. Field visits were conducted by the Registry Coordinators (RC) at the medical records offices of restructured and private hospitals for data collection. Data extractions were done in accordance to the NBDR guidelines and all data were entered directly and secured in the database. Annual audits were done to standardise the definitions of data items and abstraction rules to ensure standards of consistency and accuracy in data collection. Inter-rater reliability (IRR) audits of similar cases abstracted by multiple RCs were checked for levels of agreement and the kappa coefficient ≥ 0.95.

The International Classification of Diseases 9th Edition (ICD-9) with Extension of the British Paediatric Association (BPA) Classification of Diseases (1979) Coding of Birth Defects^33^ was used for subjects registered from 2003 to 2012. Individuals registered from 2012 onwards were coded using the International Classification of Diseases 10th Edition (ICD-10) Chapter XVII Royal College of Paediatric Child and Health Extension^34^. Extractions from the Registry’s database were done by using the following codes in ICD-9: 749, and in ICD-10: Q35–Q37. Cleft laterality (side of the cleft), submucous cleft, bifid uvula and severity grading of the cleft defects were not classifiable by the ICD-9 and ICD-10 codes and they could not be recorded.

The count of infants with clefts was by live pregnancy outcomes of Singaporean mothers grouped by ethnicity and who were citizens or permanent residents living in Singapore in the period 2003 to 2012. Live births of foreigners in Singapore and Singaporeans who did not reside in the country were excluded. Stillbirths and abortions (spontaneous and elective) were also excluded. The denominator was population live births per 10,000. Statistical analysis was performed using the SAS version 9.3 (SAS Institute, Cary, NC, USA). Linear regression was used in trend tests and the significance level was set at p < 0.05.

The Strengthening the Reporting of Observational Studies in Epidemiology (STROBE) checklist for cohort studies^35^ was used to guide the reporting of this study in identifying at-risk groups of cleft lip and/or palate development, one of the non-communicable disease conditions that posed major health burdens. The goals were to promote a health information system and surveillance to show the scale and impact for current patterns of health and disease in infants born with cleft lip and/or palate.

### Ethics approval

The ethics of the study was approved by the Singapore National Birth Defects Registry Office (Reference: Y15-007) and the SingHealth Centralised Institutional Review Board D (Reference: 2014/2199) with waiver of informed consent as secondary data were used.

## Data Availability

Data supporting this study are at: https://osf.io/5rw3f/files/.
